# A cellulose acetate/*Amygdalus pedunculata* shell-derived activated carbon composite monolith for phenol adsorption†

**DOI:** 10.1039/c7ra13017a

**Published:** 2018-02-16

**Authors:** Qiancheng Xiong, Qiuhong Bai, Cong Li, Yuanyuan He, Yehua Shen, Hiroshi Uyama

**Affiliations:** Key Laboratory of Synthetic and Natural Functional Molecule Chemistry of Ministry of Education, College of Chemistry and Materials Science, Northwest University Xi'an 710127 Shaanxi Province China; College of Pharmaceutical Engineering, Shaanxi Fashion Engineering University Xi'an 712046 Shaanxi Province China; Department of Applied Chemistry, Graduate School of Engineering, Osaka University Suita 565-0871 Japan uyama@chem.eng.osaka-u.ac.jp

## Abstract

*Amygdalus pedunculata* is expected to be a good candidate plant for desert reclamation (“greening”) since it has notable tolerance to cold and drought and can grow in a wide range of areas with different soil types and moisture contents. In this study, we have developed a single-step method to fabricate a cellulose acetate (CA)/*A. pedunculata* shell (APS)-derived activated carbon (AC) composite monolith by thermally induced phase separation (TIPS) for removal of toxic phenol from aqueous solution. The composite monolith was easily fabricated by TIPS of a CA solution in the presence of the dispersed AC, in which AC was well loaded onto the monolithic skeleton of CA. The as-obtained monolith showed a maximum adsorption capacity of 45 mg g^−1^ at the initial phenol concentration of 0.8 mg mL^−1^. The present composite can be prepared with an arbitrary shape by a facile method from cheap materials, and is more convenient to recycle than powder adsorbents. Therefore, the present CA/APS-derived AC composite monolith has great potential as a promising adsorbent of low cost with convenient separation for toxic phenol-containing wastewater.

## Abstract 2

In this study, we have developed a single-step method to fabricate a cellulose acetate (CA)/APS-derived activated carbon (AC) composite monolith by thermally induced phase separation (TIPS) for removal of toxic phenol from aqueous solution.

## Graphical abstract

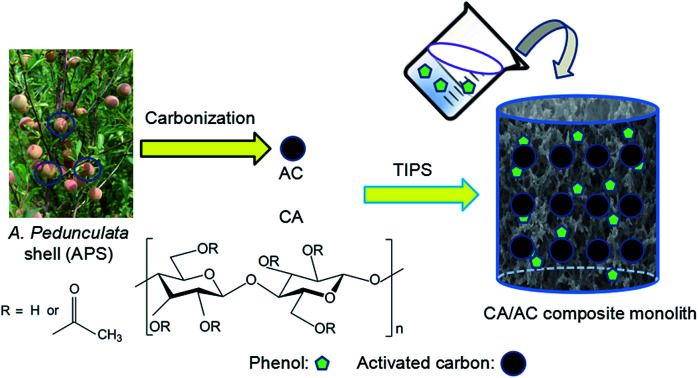

## 1. Introduction

Polymer-based monoliths, which emerged in the early 1990s, have a unique open-cellular three-dimensional (3D) interconnected porous structure. They have many applications such as chromatography, catalysis and immobilization of biomolecules, and they are considered an outstanding functional material for various uses.^1–3^ Polymer monoliths can be fabricated either by polymerization or phase separation.^4–6^ We have developed fabrication methods for polymer monoliths by thermally-induced phase separation (TIPS) or nonsolvent-induced phase separation of a polymer solution toward a variety of polymers.^7–10^ For cellulose acetate (CA), a TIPS method using a mixture of *N*,*N*-dimethylformamide (DMF) and 1-hexanol as phase separation solvent gave a monolith with a hierarchically porous structure.^1,11^

In recent years, cellulose, which is the most important forestry resource, has been converted into high-value products, and applications of cellulose have attracted great attention from both academia and industry due to shortage of fossil fuels. Among cellulose derivatives, CA is one of the most widely used cellulose-based plastics, and its typical applications are paints, textile fibers, cigarette filters, packaging materials, films and reverse osmosis membranes.^12,13^

AC is widely used in a variety of industrial applications including as adsorbents, catalyst supports, separation media, electrodes for batteries and fuel cells, and materials for hydrogen and carbon dioxide storage.^14–16^ Some of AC are produced from biomass by carbonization and activation processes. *Amygdalus pedunculata* is a perennial, sand dune-stabilizing, and oil-bearing shrub. *A. pedunculata* has notable tolerance to cold and drought, and can grow in a wide range of areas with different soil types and moisture contents. Thus, it is expected to be a good candidate for desert reclamation in China. Recently, we have prepared AC from *A. pedunculata* shells (APS) and evaluated its adsorption and electrochemical performance.^17,18^

Main source of fresh water pollution is discharge of untreated sanitary and toxic industrial wastes. Phenol, which is difficult to degrade in nature, is a common and highly toxic organic pollutant that is extensively found in coking residues, papers, gases, oil refining wastes and other industrial waste liquids.^19^ Human poisoning by phenol is caused mainly through contact with respiratory tract and skin.^20^ The low concentration induces protein denaturation, and the high concentration causes protein precipitation, which results in direct damage to cells and paralysis of central nervous system. Therefore, effective removal of phenol from wastewater is an important research issue, and the research outcomes could lead to development of wastewater treatment.

AC powder is a common adsorbent that demonstrates good performance for adsorption of phenol in wastewater because of its well-developed internal pore structure and high surface area.^21,22^ However, it is difficult and often costly to separate from wastewater,^23^ and thermal regeneration may have environmental and economic constraints. Recently, porous polar polymer adsorbents for adsorption of phenol were developed, however, they were also difficult to separate in a continuous flow system.^24^ In this study, we therefore propose a composite monolith consisting of a porous polymer and AC for efficient removal of phenol that combines advantages of monolithic materials and AC. Fabrication of such a composite monolith *via* polymerization of vinyl monomers in the presence of AC is a possible pathway, but difficult in most cases because AC acts as an inhibitor for radical polymerizations. Additionally, dispersants often affect monolith morphology, and this preparation method is time-consuming.

In this study, we designed a three-dimensional porous composite material from CA and AC for convenient and efficient removal of phenol from wastewater. The CA/AC composite monolith was prepared by TIPS of CA in the presence of APS-derived AC, and its phenol adsorption ability was evaluated. To date, CA membranes for separators and adsorbents were reported to be prepared by electrospinning and phase separation techniques,^25,26^ but a single-step preparation of CA/inorganics composite membranes has rarely been studied. This study provides a facile single-step fabrication route for such porous composites, which is preferable in practical environmental applications. The dispersed compound can be easily immobilized on the skeleton surface of the monolith polymer, and both of the size of flow-through channels and the pore depth are tuned by the fabrication parameters. Furthermore, the monolith can be prepared with an arbitrary shape according to actual demands, and recovery of the monolithic adsorbent is very convenient.

## 2. Experimental

### 2.1 Materials and reagents

CA with a molecular weight of 5 × 10^4^ (acetyl content of 39.3–40.3 wt%) was obtained from Sigma-Aldrich Co. Phenol, DMF, dimethyl sulfoxide (DMSO), ethanol and sodium hydroxide (NaOH) were purchased from Tianjin Zhiyuan Chemical Industries, Ltd. 1-Hexanol and 1-butanol were obtained from Tianjin Guangfu Fine Chemical Research Institute. All reagents were of analytical grade and were used as received without further purification. APS-derived AC was prepared in our laboratory according to the literature.^17^

### 2.2 Characterization

Morphologies of the CA monolith and CA/AC composite monolith were observed using a scanning electron microscope (SEM). SEM images were recorded on a HITACHI S-3000N instrument at 15 kV. A thin gold film was sputtered on the samples under vacuum before images were collected. Nitrogen adsorption/desorption isotherms were measured with a TR2 Star3020 surface area pore size analyzer at 77 K. Before analysis, the samples (at least 50 mg) were degassed at 373 K under vacuum for at least 8 h. Specific surface area and pore size distribution (PSD) were determined by Brunauer Emmett Teller (BET) equation and non-local density functional theory (NLDFT) method, respectively. Thermogravimetric analysis (TGA) was performed with a NETZSCH STA 449C thermogravimetric analyzer from 33 °C to 900 °C at a heating rate of 10 °C min^−1^ under a steady nitrogen flow of 300 mL min^−1^. UV-vis absorbance was measured at a wavelength of 268 nm using a SHIMADZU UV-1700 spectrophotometer.

### 2.3 Fabrication of CA/AC porous composite monolith

Fabrication of the CA/AC composite monolith was performed by TIPS of a CA solution in the presence of AC. CA powder (2.0 g) was dissolved completely in DMF (10 mL) at 70 °C, and 1-hexanol (10 mL) was added dropwise to the CA solution in two parts to prevent the generation of precipitates. AC (1.0 g) and 1-hexanol (5 mL) were added under gentle stirring. The solution was kept at 70 °C for 12 h to form a good dispersion, and it stood at room temperature for additional 12 h to complete the phase separation. The solvent was replaced with ethanol at least three times and subsequently dried under vacuum to give the CA/AC composite monolith.

### 2.4 Adsorption test

#### 2.4.1 Effect of contact time

Twenty mg of the CA monolith or CA/AC composite monolith (CA/AC = 2/1 and 1/1 w/w) was added to 25 mL of a phenol solution (initial concentration of 0.25 mg mL^−1^), and the mixture was kept under gentle shaking of 180 rpm at 25 °C in a thermostat shaker. At various absorption times (2, 4, 6, 8, 10, 20, 30, 40, and 50 h), the concentration of the residual phenol was measured by a UV-vis spectrophotometer after the monolithic sample was removed. The phenol amount adsorbed by the CA/AC composite monolith was calculated according to eqn (1):

1 *q*_e_ = (*C*_0_ − *C*_*t*_)*V*/*m*

where *q*_e_ is the amount of equilibrium adsorption (mg g^−1^) and *C*_0_ and *C*_*t*_ are the initial and equilibrium adsorption concentrations in solution at different contact times with phenol (mg mL^−1^). *V* is the volume of aqueous phenol solution, and *m* is the monolith weight (g). For each sample, the experiment repeated at least three times.

#### 2.4.2 Effect of initial concentration

Aqueous phenol solutions (25 mL) at concentrations of 0.1–1.0 mg mL^−1^ were prepared. The adsorption experiment was performed using 0.02 g of CA/AC composite monolith for 30 h under gentle shaking of 180 rpm at 25 °C. For each sample, the experiment repeated at least three times.

#### 2.4.3 Effect of pH

The desired pH of the solution was varied from 2 to 11 by adding dilute hydrochloric acid or sodium hydroxide. The CA/AC composite monolith (0.02 g) was added to the phenol solution of the concentration of 0.25 mg mL^−1^ with varying pH, and after the adsorption equilibrium, the amount of equilibrium adsorption was determined similarly. For each sample, the experiment repeated at least three times.

#### 2.4.4 Desorption and regeneration

The CA/AC composite monolith (0.2 g) was added to 25 mL of phenol solution with concentration of 0.8 mg mL^−1^, and after the equilibrium was reached, the monolith was dried under vacuum. The dried monolith was immersed in NaOH solution (2 wt%, 50 mL), and the amount of phenol in the solution at different times was determined.

Regeneration efficiency (*Z*) was calculated according to eqn (2):

2 *Z* (%) = (25 (mL) × *C* (mg mL^−1^)/1000)/*m* (g) × 100%

where *Z* is the regeneration efficiency, *C* is the concentration of phenol in the NaOH solution, and *m* is the adsorption amount of phenol on the composite monolith. After treatment with the eluent, the adsorbents were washed with distilled water and reused in consecutive adsorption–desorption cycles. For each sample, the experiment repeated at least three times.

## 3. Results and discussion

### 3.1 Preparation and characterization of CA/AC composite monolith

**Fig. 1:**
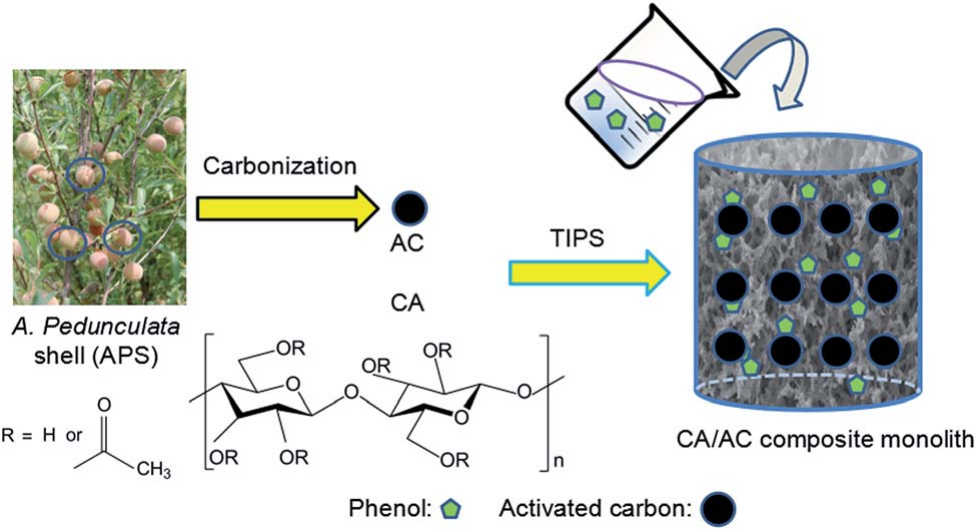
Schematic process diagram of APS utilization for phenol adsorption.

Lignocellulosic biomass is a renewable energy resource that has been reported to be a precursor for the preparation of AC.^27,28^ APS serves as typical lignocellulosic biomass collected from *A. pedunculata* plant. This plant is a good candidate for desert reclamation and can produce edible oil.^29^Fig. 1 shows a schematic process diagram of the APS utilization for phenol adsorption, and the preparation process for the CA/AC composite monolith is shown in Fig. S1.† Previously, we used commercial AC for the preparation of the similar composite monolith and hydrolyzed the side chain of CA to produce the cellulose/AC composite showing good absorption properties for toxic dyes.^11^

**Fig. 2:**
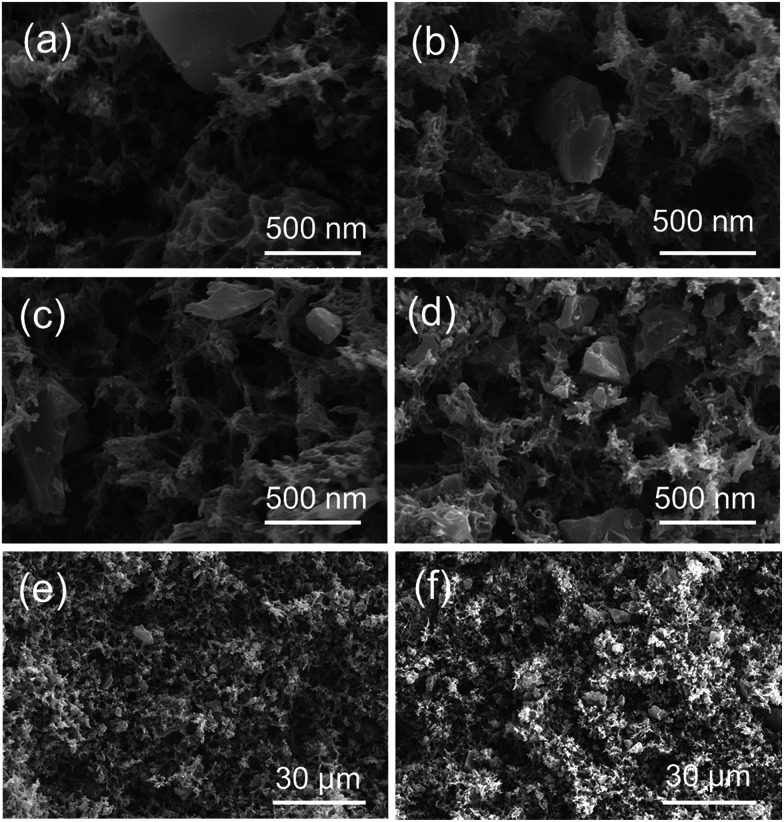
SEM images of composite CA/AC monolith with different weight ratios of (a) and (e) 4/1, (b) and (f) 2/1, (c) 4/3, and (d) 1/1 ((e) and (f): images in low magnification).

It was reported that multi-walled and multi-branched nanotubes have a high adsorptive ability for phenol compared to other nano-sized carbon materials.^30–32^ SEM observation showed a three-dimensional open-porous walled structure, which was conducive to the efficient adsorption of phenol.^33–35^ In the preparation of composite materials from dispersions, a stable and homogeneous dispersion of functional materials should be retained during the formation of polymer matrix.^24,36^ In the present study, APS-derived AC was well suspended in the CA solution of a DMF/1-hexanol mixture, resulting in the good dispersion of AC on the skeleton surface of the monolith (Fig. 2). To ensure that AC can be evenly dispersed in the CA solution, it is necessary to control the size of the AC powder and rapidly cool for the formation of colloids.

Fig. S2(a)† shows the magnified SEM image of APS-derived AC, which suggests the dense structure and small pores of pure AC. The present APS-derived AC had a maximum BET surface of 2060 m^2^ g^−1^.^17^ From SEM images of the CA monolith (Fig. S2(b)†) and the CA/AC composite monolith (Fig. 2), the formation of the macrovoid and continuous open-cellular three-dimensional (3D) interconnected porous structure was observed. In the CA/AC composite monolith, AC was found in the CA monolith matrix and the internal morphology of the monolith changed with the addition of AC. With increase of the amount of AC, more AC was embedded in the framework of the composite monolith.

**Fig. 3:**
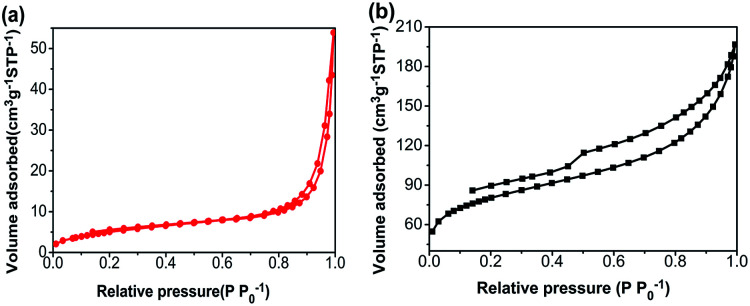
Nitrogen adsorption–desorption isotherms of (a) CA monolith and (b) CA/AC composite monolith.

**Fig. 4:**
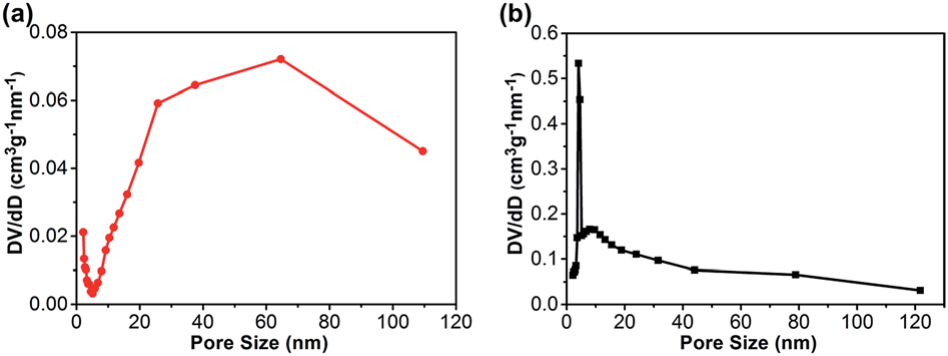
Pore-size distribution of (a) CA monolith and (b) CA/AC composite monolith.

Nitrogen adsorption–desorption isotherms of the CA monolith and the CA/APS-derived AC composite monolith are shown in Fig. 3. The CA monolith exhibited a typical V, with a type H1 hysteresis loop in the *P*/*P*_0_ range from 0.8–1.0, and the amount of adsorption drastically increased in the latter part of the isotherm (Fig. 3(a)). The composite monolith was type IV and had a hysteresis loop of type H2 at *P*/*P*_0_ 0.4–1.0, which indicates a relatively uniform mesoporous and macroporous structure (Fig. 3(b)). The presence of these mesopores and macropores enables the wider potential applications of the present composite monolith in industrial fields. The corresponding pore-size distribution in the range of mesopores (2–50 nm) (Fig. 4(a)) and pore diameter of the CA/AC composite monolith was centered at 4 nm (Fig. 4(b)). The specific surface area of the CA monolith was 19 m^2^ g^−1^, and the CA/AC composite monolith possessed a much larger BET surface area of 262 m^2^ g^−1^ (Table S1†). This result suggests that the CA/AC composite monolith will have a better adsorption performance than the CA monolith.

The present composite can facilitate the diffusion of solution into the large internal network of spaces. Therefore, the increased contact area between the composite and solution promotes the adsorption of phenol. The composite monolith can be prepared with an arbitrary shape, which also advances its practical use compared to the powder form of AC.

**Fig. 5:**
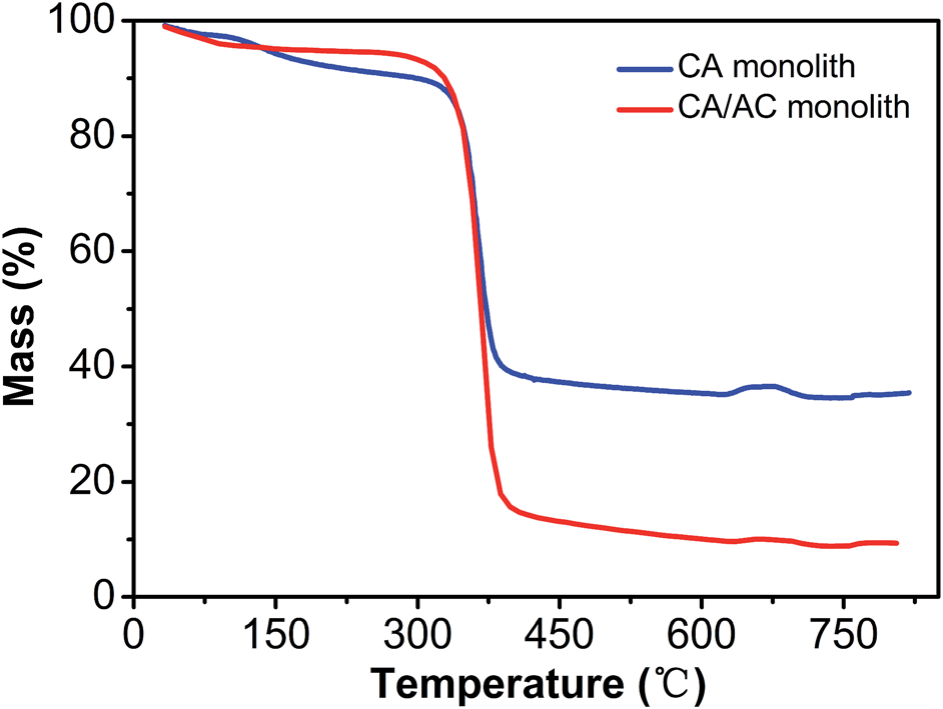
TG curves of CA monolith and CA/AC composite monolith.

Thermal stability of the obtained monolith was analyzed by TGA (Fig. 5). The CA monolith demonstrated an obvious weight loss from 350 °C to 410 °C. The CA/AC composite monolith had three thermal decomposition stages, which were observed at 100–200 °C, 200–300 °C, and 300–430 °C. The first phase of the weight loss might be the loss of water. The weight loss at 200–300 °C was due to the decomposition of cellulose acetate. The weight loss of the CA monolith reached 90% after the thermal treatment, whereas approximately 60% of the CA/AC composite monolith remained even at 390 °C. This difference indicates that the present composite showed better thermal stability than the CA monolith alone, because of the high thermal stability of AC.

### 3.2 Adsorption of phenol

**Fig. 6:**
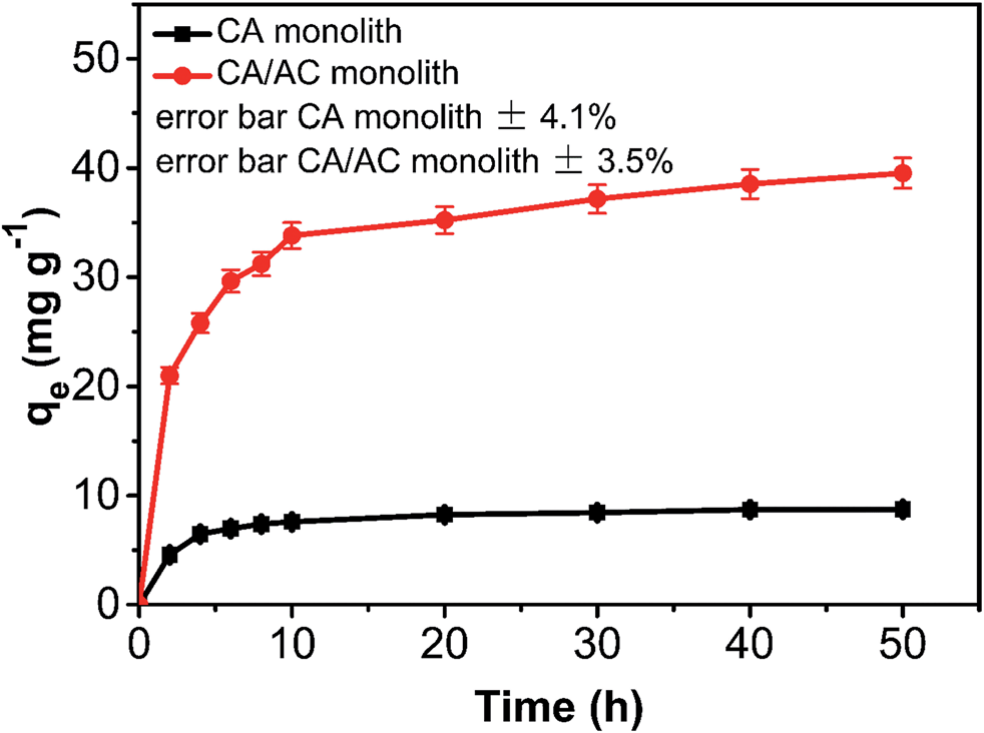
Phenol adsorption on CA monolith and CA/AC composite monolith at various times (*C*_0_: 0.8 mg mL^−1^, pH: 7, adsorbent dosage: 0.02 g, temperature: 25 °C, CA : AC, 1 : 1).

**Fig. 7:**
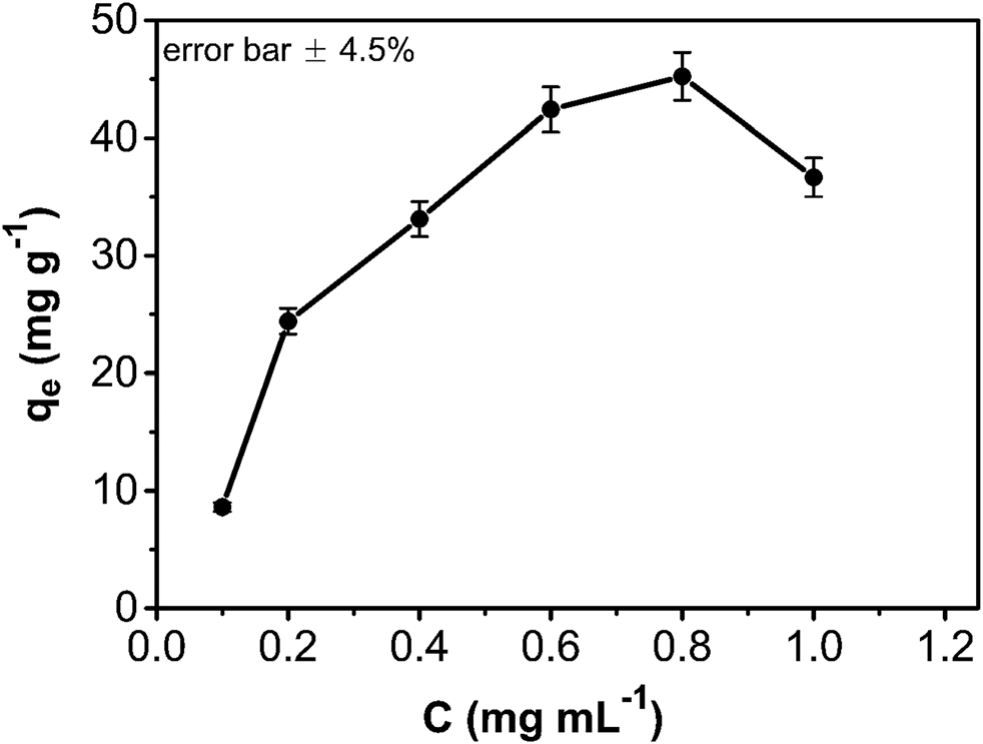
Effect of contact time and initial absorption concentration on CA/AC composite monolith (pH: 7, adsorbent dosage: 0.02 g, temperature: 25 °C, CA : AC, 1 : 1).

The adsorption amount of phenol onto the CA/AC composite increased quickly in the first 10 h (Fig. 6). From 10 to 30 h, the adsorption became slower, and after 30 h, the phenol adsorption nearly reached equilibrium. The adsorption kinetics is separated into three stages; the first is the initial rapid phase of the adsorption, the second is the slow adsorption stage, and the final stage is the adsorption equilibrium stage. When the phenol initial concentration was 0.8 mg mL^−1^, the adsorption capacity of the CA/AC composite monolith was 41 mg g^−1^, whereas that of the CA monolith was only 9 mg g^−1^. With increased proportion of AC, the adsorbed amount of phenol increased. When the amount of AC was the same as that of CA, the maximum adsorption capacity was found (Fig. S3†). The preparation of the composite monolith with the well dispersion of AC was difficult in the further increase of the AC amount. As shown in Fig. 7, the adsorption amount of phenol on the composite monolith increased from 8 mg g^−1^ to 45 mg g^−1^ at various initial concentrations. The maximum capacity adsorption of the composite monolith was 45 mg g^−1^, with an initial concentration of phenol 0.8 mg mL^−1^.

Phenol removal was well correlated with the specific BET surface; the higher specific BET surface of the composite resulted in the higher removal efficiency of phenol. Generally, hydroxyl and carbonyl groups play pivotal roles in the adsorption process, which may be primarily facilitated by hydrogen bonding between the abundant carbonyl and hydroxyl groups of CA surface^37^ and the good adsorption capacity of AC.^26^ The electrons of the phenol ring and the electrons of the graphene layer in the activated carbon induce aromatic–aromatic interactions when the adsorption takes place. In addition to AC in the composite, the molecular structure of phenol should be considered; the partially positive hydrogen atom of phenol forms the hydrogen bonding with the carbonyl groups of CA monolith more readily. Based on these reasons, the CA/AC composite monolith possessed better adsorption for phenol than the CA monolith at the same contact time.

**Fig. 8:**
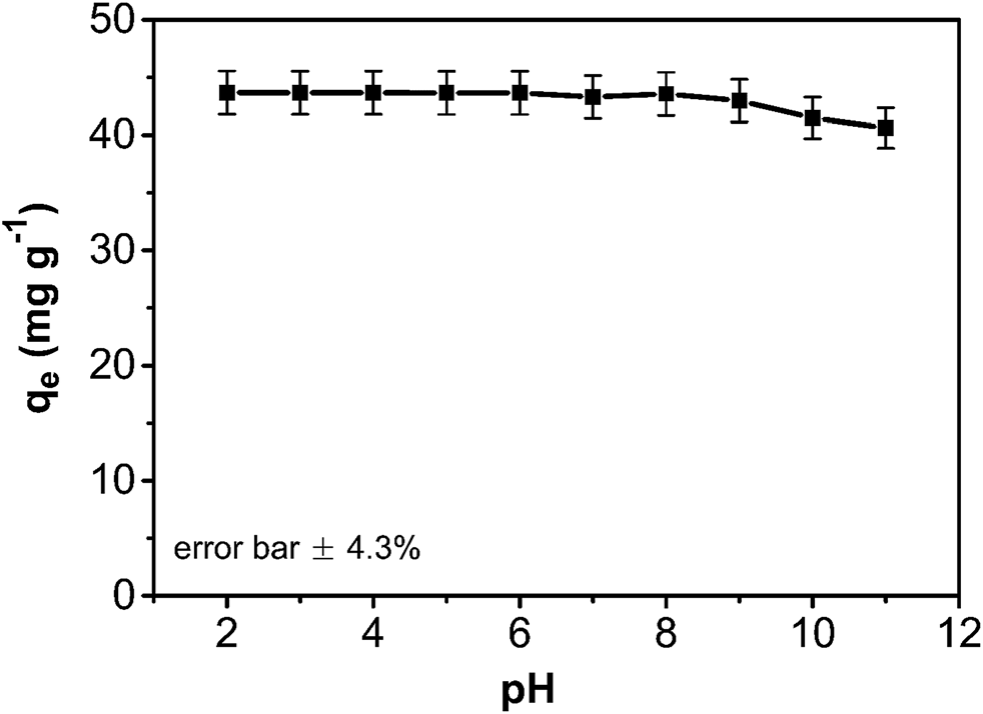
pH effect on adsorption capacity of CA/AC composite monolith (*C*_0_: 0.8 mg mL^−1^, adsorbent dosage: 0.02 g, temperature: 25 °C, CA : AC, 1 : 1).

As an important variable parameter, the effect of pH on the phenol adsorption by the composite was investigated (Fig. 8). The adsorption capacity of the CA/AC composite monolith was not sensitive to pH changes from 2.0 to 9.0 but slightly declined at pH above 9.0. This observation could be explained by the potential effect of pH variation on the phenol adsorption process. Phenol is a weak organic acid (p*K*_a_ = 9.98) that exists in the protonated form in acidic and neutral conditions and an ionized form in the alkaline condition. As pH increases, the surface charge density of the composite becomes more negative and more phenol molecules are deprotonated. As a result, the electrostatic repulsion between the surface composite and negatively charged phenol molecules hinders the adsorption.

**Fig. 9:**
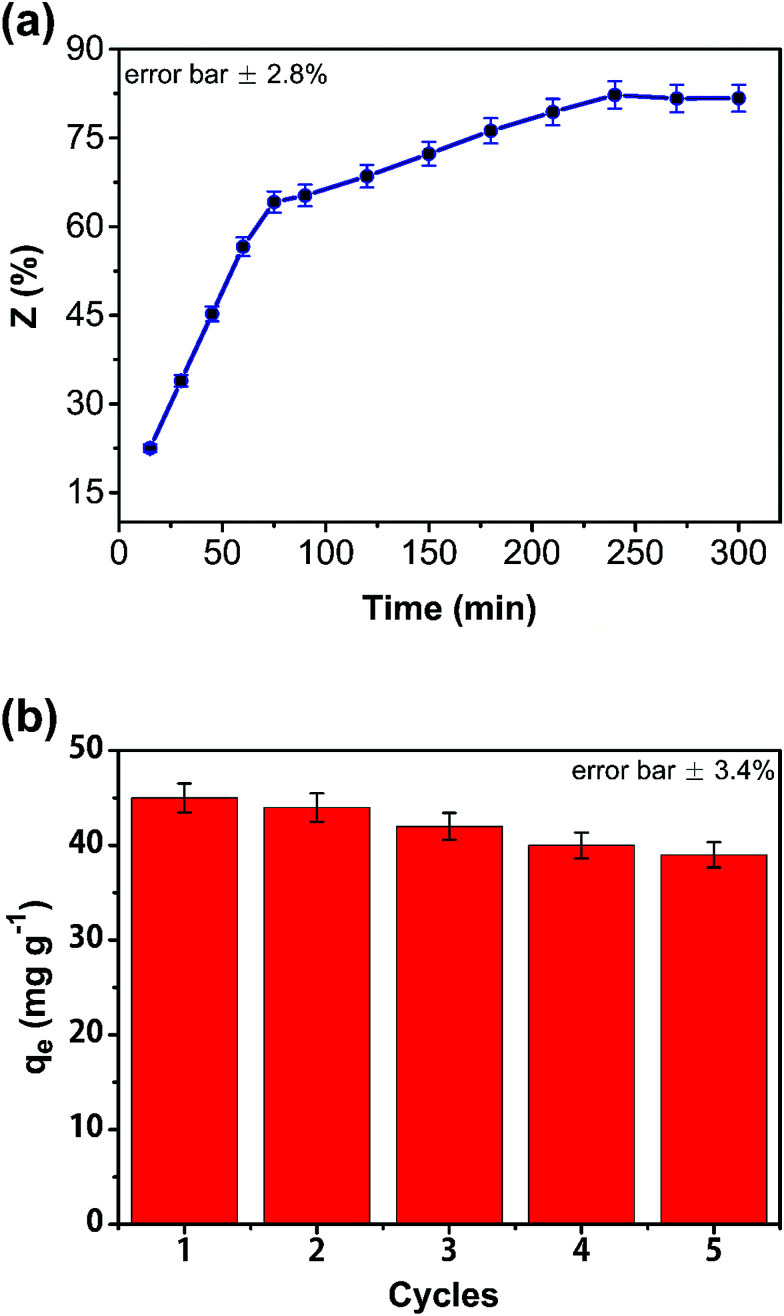
Regeneration and repeated use of CA/AC composite (*C*_0_: 0.8 mg mL^−1^, pH: 7, adsorbent dosage: 0.02 g, temperature: 25 °C, CA : AC, 1 : 1).

The effect of eluent on regeneration was investigated (Fig. 9(a)). Sodium hydroxide was used for desorption of phenol from the composite monolith. Regeneration efficiency (*Z*) was calculated from the desorption amount of phenol. In the first 75 min, the desorption rate of phenol in the composite was very rapid; 82% of the phenol was desorbed from the CA/AC composite monolith after 3 h, indicating that sodium hydroxide could effectively induced the removal of phenol from the monolith surface with macropores and mesopores. To examine the stability of this adsorbent, adsorption–desorption cycles were examined. After 5 cycles, the adsorption capacities slightly decreased from 37 to 33 mg g^−1^ (Fig. 9(b)). Therefore, the obtained adsorbent was found to have stable physical and chemical properties and be able to be regenerated for 5 times.

## 4. Conclusions

A CA/AC composite monolith with three-dimensionally interconnected porous structures was successfully fabricated in a single step by TIPS, which can remove phenol in high efficiency from an aqueous solution. The preparation method of the composite monolith was simple and convenient, and can more evenly distribute AC on the skeleton of the CA monolith. Additionally, the present study provides a new approach for the utilization of APS, contributing to application development for greening plants. As a whole material, the composite monolith was more convenient to recycle than the powder adsorbents. The maximum capacity adsorption of the composite monolith was 45 mg g^−1^ at an initial phenol concentration of 0.8 mg mL^−1^. The adsorption property and thermal stability of the composite were much better than those of the CA monolith. It can be concluded that the CA/AC composite is a new class of phenol adsorbents. Further studies on the fabrication of composite monoliths from a variety of combinations of common polymers and functional inorganics as well as the applications of such monoliths are underway in our laboratory.

## Conflicts of interest

There are no conflicts to declare.

## Supplementary Material

RA-008-C7RA13017A-s001
