# Supplementary material for: A cellulose acetate/Amygdalus pedunculata shell-derived activated carbon composite monolith for phenol adsorption
Source: RSC Adv. 2018 Feb 16;8(14):7599–605. doi: 10.1039/c7ra13017a (PMC9078407; doi:10.1039/c7ra13017a)
Supplement: RA-008-C7RA13017A-s001 [file RA-008-C7RA13017A-s001.pdf]

Electronic Supplementary Material (ESI) for RSC Advances.
This journal is © The Royal Society of Chemistry 2018

Supporting Information

Cellulose acetate/*Amygdalus pedunculata* shell-derived activated carbon composite monolith for phenol adsorption

Qiancheng Xiong[a,b], Qiuhong Bai[a], Cong Li[a], Yuanyuan He[b], Yehua Shen†[a], Hiroshi Uyama†[a,c]

[a] Key Laboratory of Synthetic and Natural Functional Molecule Chemistry of Ministry of Education, College of Chemistry and Materials Science, Northwest University, Xi'an 710127, Shaanxi Province, China

[b] College of Pharmaceutical Engineering, Shaanxi Fashion Engineering University, Xi'an 712046, Shaanxi Province, China

[c] Department of Applied Chemistry, Graduate School of Engineering, Osaka University, Suita 565-0871, Japan

Corresponding author: Tel: +86-29-88302635; E-mail: yhshen@nwu.edu.cn

Corresponding author. Tel.: +81-6-6879-7364; Fax: +81-6-6879-7367;

E-mail: uyama@chem.eng.osaka-u.ac.jp

Content:

Fig. S1. Fabrication procedure of the CA/AC composite monolith

Fig. S2. SEM images of AC power (a), CA monolith(b)

Fig. S3. Effect of different AC power (m CA : AC) adsorption onto CA/AC composite monolith (C_o: 0.8 mg/mL, pH:7, adsorbent dosage: 0.02 g, temperature: 25 ^oC)

Table S1. Surface properties of CA monolith and CA/AC monolith

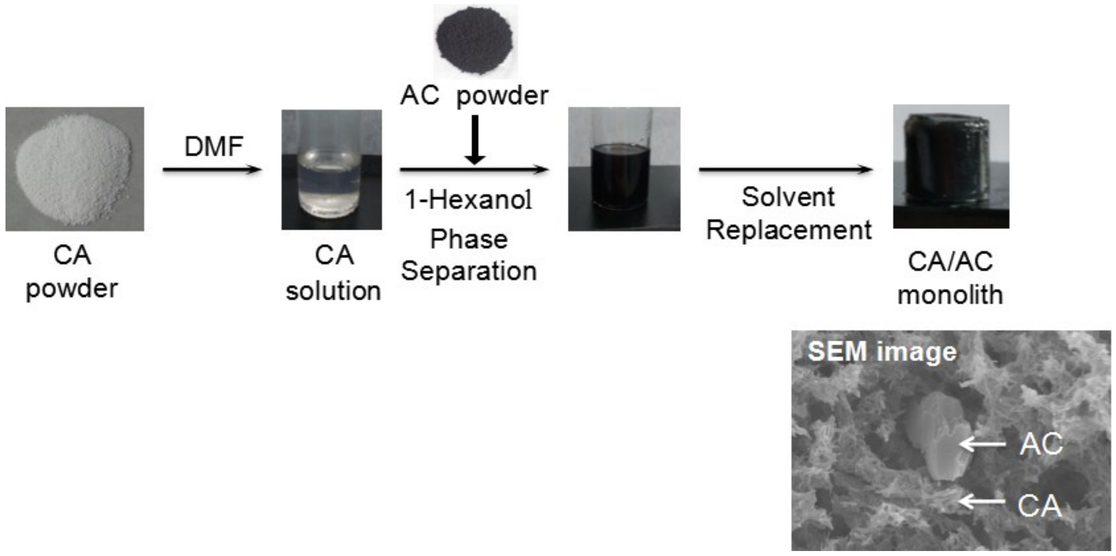

Fig. S1. Fabrication procedure of the CA/AC composite monolith

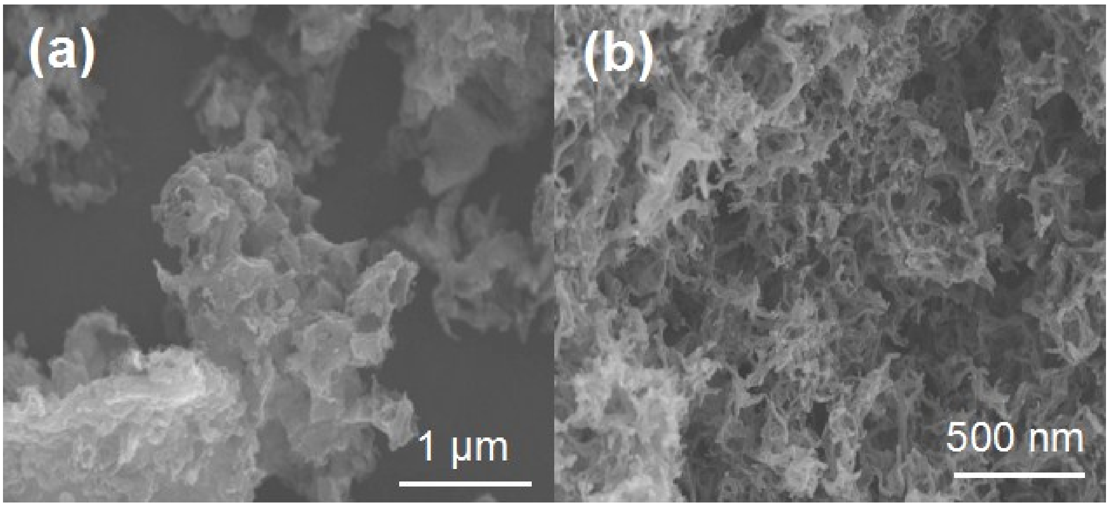

Fig. S2. SEM images of AC power (a), CA monolith(b)

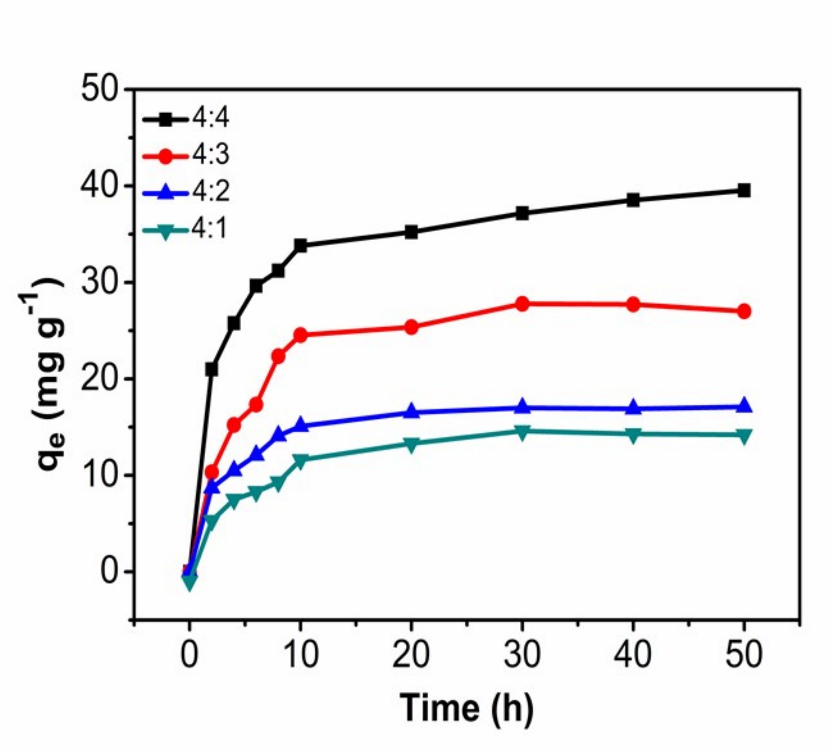

Fig. S3. Effect of different AC power (CA : AC) adsorption onto CA/AC composite monolith (C_o: 0.8 mg/mL, pH:7, adsorbent dosage: 0.02 g, temperature: 25 ^oC)

Table S1. Surface properties of CA monolith and CA/AC monolith

Samples	Surface Area ($m^2 g^{-1}$)	Pore Volume (cm^3/g)	Pore Size (nm)
CA monolith	19	0.04	9.1
CA/AC monolith	262	0.26	4.0
